# Quasiclassical Trajectory Study of NH(^3^Σ^–^) + NH(^3^Σ^–^) Reactive Collisions: The Role of Vibrational Energy

**DOI:** 10.1021/acs.jpca.6c06053

**Published:** 2026-10-27

**Authors:** Delvany G. de Castro, Lucas W. S. Crispim, Maikel Y. Ballester

**Affiliations:** Departamento de Física, 28113 Universidade Federal de Juiz de Fora, 36036-900 Juiz de Fora, Minas Gerais, Brazil

## Abstract

We present a quasiclassical trajectory study of the reaction NH­(^3^Σ^–^) + NH­(^3^Σ^–^) → N_2_ + H + H to investigate the role of *vibrational* excitation of the reactants. Using the global six-dimensional DMBE potential energy surface for the singlet ground state of N_2_H_2_ (Poveda, L. A.; etal. J. Chem. Phys., 2009, 131, 044309). we characterize the singlet-surface contribution to this reaction. We ran trajectories for NH­(*v*, 1) colliding with NH­(*v*′, 1), with *v* = 1–5, covering both the case where a single reactant carries vibrational excitation (*v*′ = 0) and the case where both do (*v* = *v*′), for translational energies 0.1 ≤ *E*_tr_/kcal mol^–1^ ≤ 10. As in our previous study, the dominant product channel is N_2_ + H + H, formed via competing one-step and two-step (N_2_H-intermediate) microscopic mechanisms. At low translational energy, states in which both reactants are vibrationally excited show a larger two-step contribution than the corresponding singly excited states. A comparison of three states with nearly matched total vibrational energy, (4, 0), (3, 1), and (2, 2), shows that the distribution of vibrational energy between the reactants affects the mechanism branching: above a few kcal mol^–1^, sharing the excitation between both reactants favors the one-step mechanism, whereas the enhanced two-step contribution at the lowest collision energies is specific to (2, 2). The vibrational energy content of the nascent N_2_ increases markedly with reactant vibrational excitation. Reactive cross sections, excitation functions, and rate constants are also reported.

## Graphical abstract

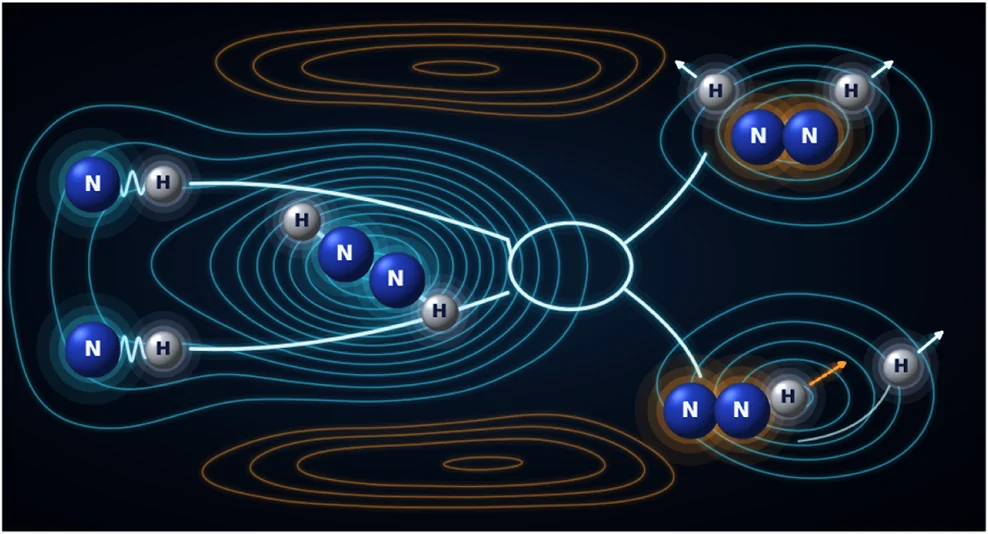

## Introduction

Despite its short lifetime as a free species, the NH radical is an intermediate in combustion chemistry and in the upper atmosphere.^1–4^ Because its ground electronic state is a triplet, ^3^Σ^–^, two colliding NH radicals can couple their unpaired electron spins in three ways, giving rise to singlet, triplet, and quintet potential energy surfaces (PESs) for the NH···NH dimer. Only the quintet surface has a genuine barrier;^5^ it correlates adiabatically with N­(^4^ *S*) + NH_2_(^2^ *A*″). The singlet and triplet surfaces instead connect, without a barrier, to a bound N_2_H_2_ intermediate.^6^ NH···NH chemistry is also relevant to cold- and ultracold-molecule research: NH has been proposed as a direct-cooling precursor, which avoids the need to associate precooled atoms.^7,8^ In that context, reactive loss channels compete with the spin-changing inelastic collisions of interest, which has motivated several sub-Kelvin NH + NH scattering studies.^5,7–9^ At high temperatures (2100–2500 K), Mertens et al.^10^ identified NH + NH as an important NH-removal step in HNCO shock-tube pyrolysis. Klippenstein et al.^6^ later revisited the same reaction in the context of NH_2_OH thermal decomposition, distinguishing a higher-barrier abstraction path (NH + NH → N + NH_2_) from a barrierless addition path (NH + NH → NNH + H).

In earlier work,^11^ the quasiclassical trajectory (QCT) method was used to study the reaction

1 $$\mathrm{NH}+\mathrm{NH}\to N_{2}+H+H$$

on the singlet PES of N_2_H_2_.^1^ That work focused on how the reactants’ *rotational* energy affects the reaction cross section, the microscopic mechanism, and the thermal rate constant, as in earlier QCT studies of rotational- and vibrational-energy effects on other triatomic and tetra-atomic exchange reactions.^12–16^ That study found that the dominant product is N_2_ + H + H, formed through two distinct microscopic pathways: a one-step mechanism, in which both hydrogen atoms leave the four-body complex simultaneously, and a two-step mechanism, in which one hydrogen atom departs first, leaving a short-lived N_2_H intermediate that subsequently loses the second hydrogen atom. (The roaming-like character of related microscopic mechanisms has been discussed extensively for other systems.^17–19^ ) Increasing the reactants’ rotational excitation systematically decreased the reaction cross section.

We now turn to *vibrational* energy. Exploratory calculations in the original study, restricted to *v*, *v*′ = 0, 1, suggested that vibrational excitation affects reactivity much less than rotational excitation does. Here we extend that exploration to *v* = 1–5, for both the asymmetric case where only one reactant is vibrationally excited and the symmetric case where both are, keeping the rotational quantum number fixed at *j* = *j*′ = 1 throughout. The question is whether the balance between the one-step and two-step microscopic mechanisms—previously found to be roughly constant (∼75%/25%) across translational energies for the rotational ground state—is itself sensitive to vibrational excitation, and how the vibrational energy content of the product N_2_ depends on the reactants’ vibrational state. The same question was addressed for the chemically related N_2_ + H_2_ system in recent work by our group on its vibrational- and translational-energy dependence.^20,21^ Other recent work on this PES and on NH includes a determination of second virial coefficients for NH···NH built on the same DMBE surface^22^ and a spectroscopic characterization of NH­(*A* ^3^Π).^23^

## Potential Energy Surface

Interatomic interactions are represented by the six-dimensional double many-body expansion (DMBE) potential energy surface for the ground (singlet) electronic state of N_2_H_2_.^1,24^ The DMBE methodology, developed by Varandas and coworkers, has been applied to several few-body systems.^25–28^ It consists of a many-body expansion in which each many-body energy term is divided into two parts, ensuring proper physical behavior at long-range while the full potential accurately reproduces the *ab initio* information. Figure 1 summarizes its main features. The entrance channel NH + NH connects, without a barrier, to trans and *cis* HNNH stationary structures (an apparently barrier-free approach; see ref 29, which in turn dissociate either to N_2_H + H, without a barrier (the very short lifetime of the N_2_H intermediate itself has been characterized in ref 28, or, having overcome the TS4 barrier (∼29 kcal mol^–1^ above the N_2_H + H asymptote), to N_2_ + H_2_. As discussed in ref 1, the long-range collinear N–H···N–H approach is attractive, while perpendicular-approach geometries are repulsive (Figure 2). This difference shapes the excitation function (see the Excitation Functions subsection below).

**Figure 1:**
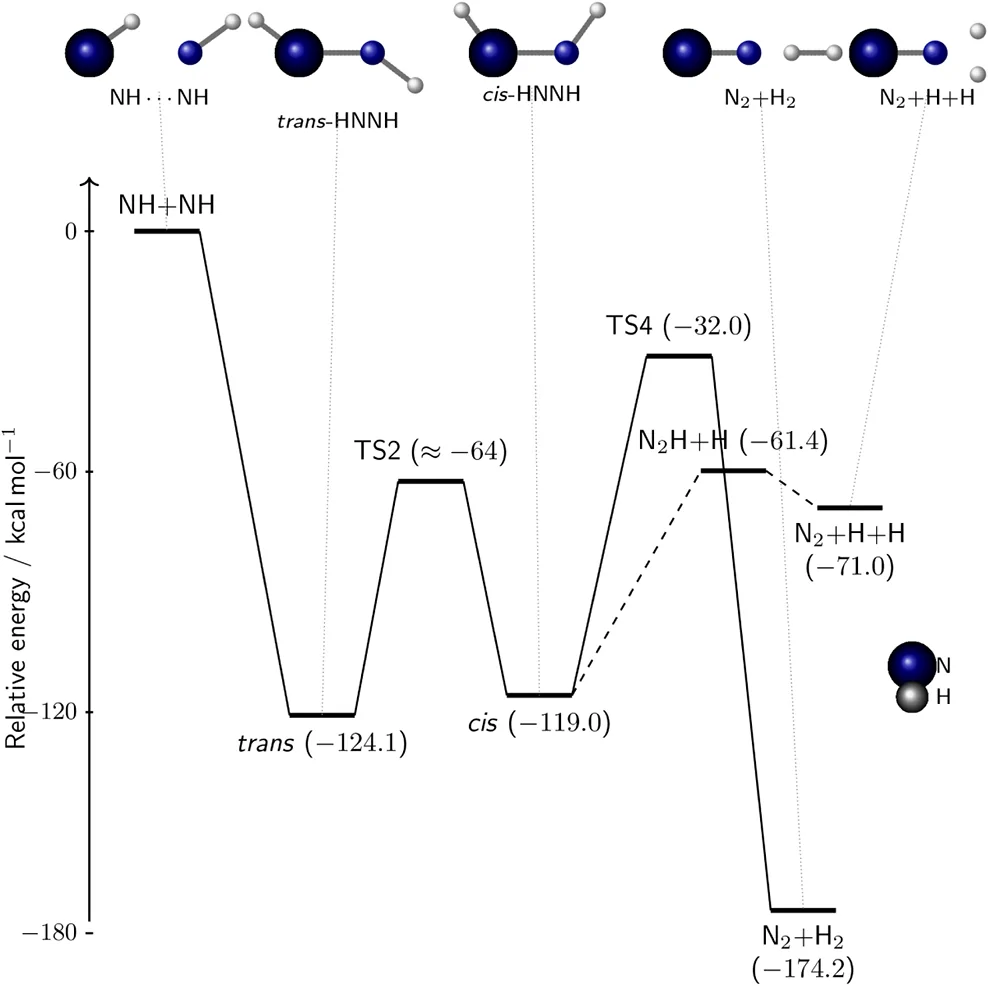
Stationary points connecting the NH + NH entrance channel to the N_2_H_2_ product channels on the singlet DMBE PES^1^ redrawn here to scale in kcal mol^–1^ (relative to NH + NH = 0) using the ZPE-corrected energies reported in ref 11, with schematic structures for the entrance channel, the trans/*cis*-HNNH minima, and the two lowest product channels. The same PES, underlies all calculations reported here; see ref 11 for the corresponding profile including the triplet and quintet surfaces.

**Figure 2:**
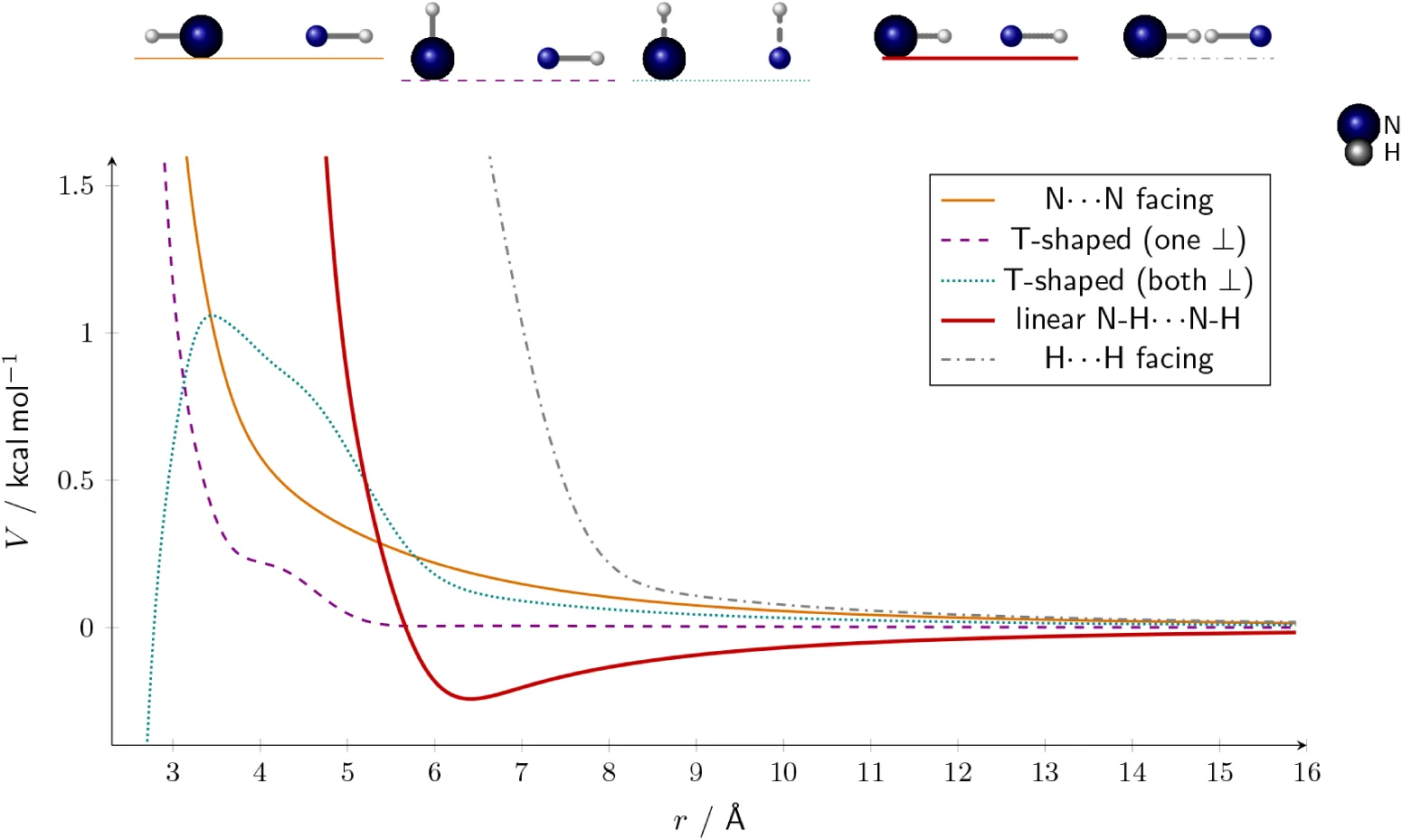
Interaction energy (relative to fully separated NH + NH) vs the N···N intermolecular distance *r*, for five relative orientations of the two rigid NH diatoms (bond length fixed at the PES’s built-in NH equilibrium value, 1.965 bohr), evaluated directly from the DMBE potential-energy function of ref 1. Only the linear, hydrogen-bridged N–H···N–H arrangement is attractive at long-range (well depth ≈ 0.23 kcal mol^–1^ at *r*  ≈ 6.4 Å); the other four orientations are repulsive or essentially noninteracting over this range, consistent with the perpendicular-approach repulsion discussed in ref 11. The H···H-facing arrangement, in which both hydrogens point toward each other across the N···N gap, is the most repulsive of the five, with no attractive well at any distance sampled.

## Calculation Details

As before,^11,20,21^ trajectories were calculated using a modified version of the VENUS96 code,^30^ which follows the general classical-trajectory methodology of refs 31 and 32 Initial ro-vibrational conditions of the NH reactants were sampled from eigenstates of the LEVEL code^33^ rather than from a Morse/harmonic guess (see ref 11). The integration step was set to 0.2 fs, and the two colliding NH fragments were initially separated by 15 Å, well beyond the range at which the NH···NH interaction becomes negligible for any of the sampled relative orientations (Figure 2). For each combination of translational energy (*E*_tr_) and reactant vibrational state (*v*, *v*′), we determined the maximum impact parameter *b*_max_ by lowering *b* from a large initial value until a reactive trajectory was obtained (accuracy of *b*_max_ ∼ 0.1 Å), and then ran *N*_T_ = 4000 trajectories for that combination, following the same reactive-cross-section prescription, σ = π*b*_max_^2^ *N*_r_/*N*_T_, introduced by Karplus, Porter, and Sharma.^34^ All 120 (*v*, *v*′) × *E*_tr_ combinations reported here were run with the full 4000 trajectories. For the additional energy-matched combination (3, 1) introduced below (12 values of *E*_tr_, 4000 trajectories each), *b*_max_ was set to the largest *b*_max_ of the neighboring combinations (1, 0)–(4, 0), (1, 1) and (2, 2) at the same *E*_tr_ plus 0.2 Å; the largest reactive impact parameter always remained below this value, and at most 1.7% of the reactive trajectories had *b* > 0.9 *b*_max_.

Trajectories reported here were propagated on the singlet electronic state of N_2_H_2_ only. The singlet surface connects the NH + NH entrance channel, without a barrier, to the deep HNNH wells and on to N_2_ + H + H. The triplet surface is also barrierless in the entrance channel: according to the *ab initio* results of Klippenstein et al.,^6^ the ^3^HNNH intermediate lies about 79.0 kcal mol^–1^ below NH + NH, and the transition state for ^3^HNNH → NNH + H lies about 52.3 kcal mol^–1^ below it. A triplet-mediated route to N_2_ + H + H is therefore energetically open, and the triplet manifold carries three times the spin-statistical weight of the singlet (*g* = 3/9 vs 1/9). To our knowledge, no global full-dimensional PES for the triplet state of N_2_H_2_ suitable for trajectory calculations is currently available. The triplet and quintet NH···NH potentials of refs,^5^,^7,8^ are four-dimensional rigid-monomer representations designed for cold and ultracold scattering, and they do not describe the reactive region. The results reported here should therefore be read as the singlet-surface contribution to NH + NH reactivity. A full-dimensional triplet PES and the corresponding dynamics are needed to establish the total NH + NH reactivity, and we leave this for future work.

The rotational quantum number was held fixed at *j* = *j*′ = 1 for both reactants throughout. This is the lowest physically meaningful value for the ^3^Σ^–^ electronic state of the NH radical (see ref 11), and this choice follows the same classical-*j* prescription validated for other Σ-state radicals colliding with closed-shell partners.^35–37^ The vibrational quantum numbers were varied over *v* = 1–5 for two sets of combinations: an *asymmetric* set, NH­(*v*, 1) + NH­(0, 1), in which only one reactant is vibrationally excited, and a *symmetric* set, NH­(*v*, 1) + NH­(*v*, 1), in which both are. For all vibrational combinations, translational energies covered the range 0.1 ≤ *E*_tr_/kcal mol^–1^ ≤ 10. Because the two sets differ in total vibrational energy as well as in its distribution between the reactants, Table 10 lists the total vibrational excitation of each combination. Several asymmetric and symmetric combinations have nearly equal totals, e.g. (2, 0) and (1, 1), or (4, 0) and (2, 2), and these pairs are used below to separate the two effects. To complete the second pair, we also ran the mixed combination NH­(3, 1) + NH­(1, 1), denoted (3, 1), whose total vibrational excitation (34.4 kcal mol^–1^) lies between those of (4, 0) and (2, 2), giving a triad of states with the same total energy but with the excitation concentrated in one reactant, partially shared, or equally shared.

As before,^11^ reactive trajectories producing N_2_ + H + H (channel 12) were classified into one-step and two-step microscopic mechanisms using the same geometric criterion: a trajectory is one-step if the N_*a*_–N_*b*_ distance remains below 3.0 Å, while the other five interatomic distances all exceed 20.0 Å, and two-step if one N–H distance exceeds 8.0 Å, while the partner N–H distance remains below 4.0 Å; every channel-12 trajectory satisfies exactly one of these two criteria. In 3.5% of all channel-12 trajectories, all of them two-step, the final arrangement assigned by the trajectory code is not N_2_ + H + H. In 3.3% the N_2_H fragment is still bound when the integration stops; given the very short lifetime of N_2_H,^28^ these are counted as N_2_ + H + H. The remaining 0.2% end as N_2_ + H_2_ (0.14%) or in channels containing an N atom or an NH fragment (0.04%), too few to affect any of the results. None of these trajectories enters the product-energy analysis below, which requires a free N_2_ fragment. Classical trajectories are susceptible to zero-point-energy (ZPE) leakage, for which several correction schemes have been proposed.^38–40^ The original rotational-energy study applied one such scheme (VEQMT) as a check on the raw QCT cross sections. Given the much larger (*v*, *v*′) × *E*_tr_ grid explored here, we take the raw QCT results as the primary statistics and give the corresponding IVEQMT-corrected result (the intermediate ZPE-leakage threshold introduced in ref 41, discussed in detail in the Reactive Cross Section subsection below) in every table and figure, so that the sensitivity to ZPE leakage can be judged.

As a check on the numerical integration, we monitored the total (kinetic + potential) energy conservation for the whole set of integrated trajectories (480 000 trajectories in total, spanning all 120 (*v*, *v*′) × *E*_tr_ combinations). Energy is conserved to better than 1 part in 1000 (0.1%) for 63% of the trajectories, and to better than 1 part in 100 (1%) for 98% of them (Figure 3). The 48 000 trajectories of the additional (3, 1) set give 67% and 98%, respectively.

**Figure 3:**
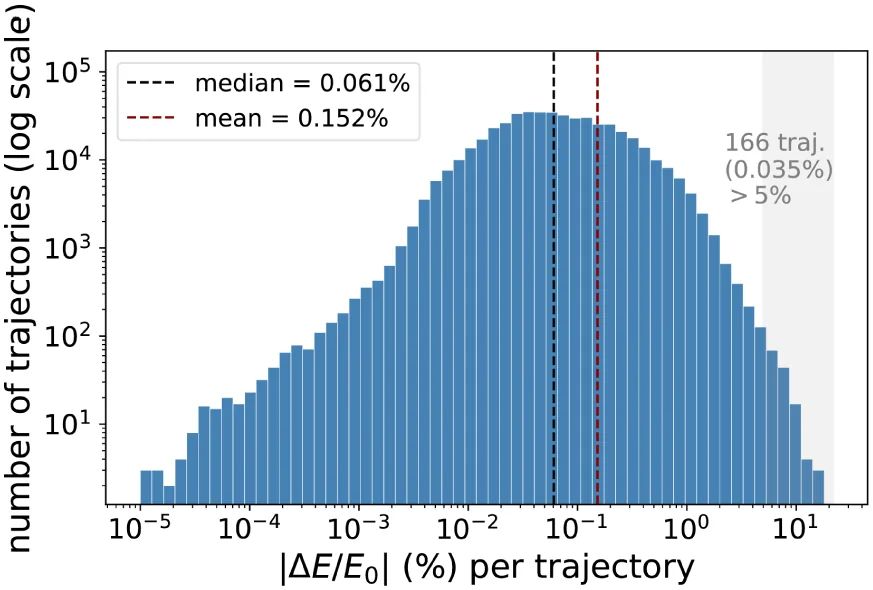
Distribution of the relative energy-conservation error, |Δ*E*/*E*_0_|, over the full production set of 480 000 trajectories (120 (*v, v*″) × *E*_tr_ combinations, *N_T_* = 4000 each; Δ*t* = 0.2 fs). The shaded region marks trajectories with |Δ*E*/*E*_0_| > 5% (166 trajectories, 0.035% of the total).

## Results and Discussion

### Microscopic Reaction Mechanisms

Figure 4 shows one representative trajectory of each type, for NH­(1, 1) + NH­(1, 1) at *E*_tr_ = 1.0 kcal mol^–1^. In the one-step case (left), all distances involving the hydrogen atoms diverge essentially simultaneously once the complex breaks apart (*t*  ≈ 1.40 ps). In the two-step case (right), one N–H distance begins to diverge first (*t*  ≈ 1.48 ps), and the second follows only after the N_2_H intermediate has survived for roughly 0.05 ps, close to the intermediate lifetime reported in ref decastro2019quasiclassical for the rotationally resolved case.

**Figure 4:**
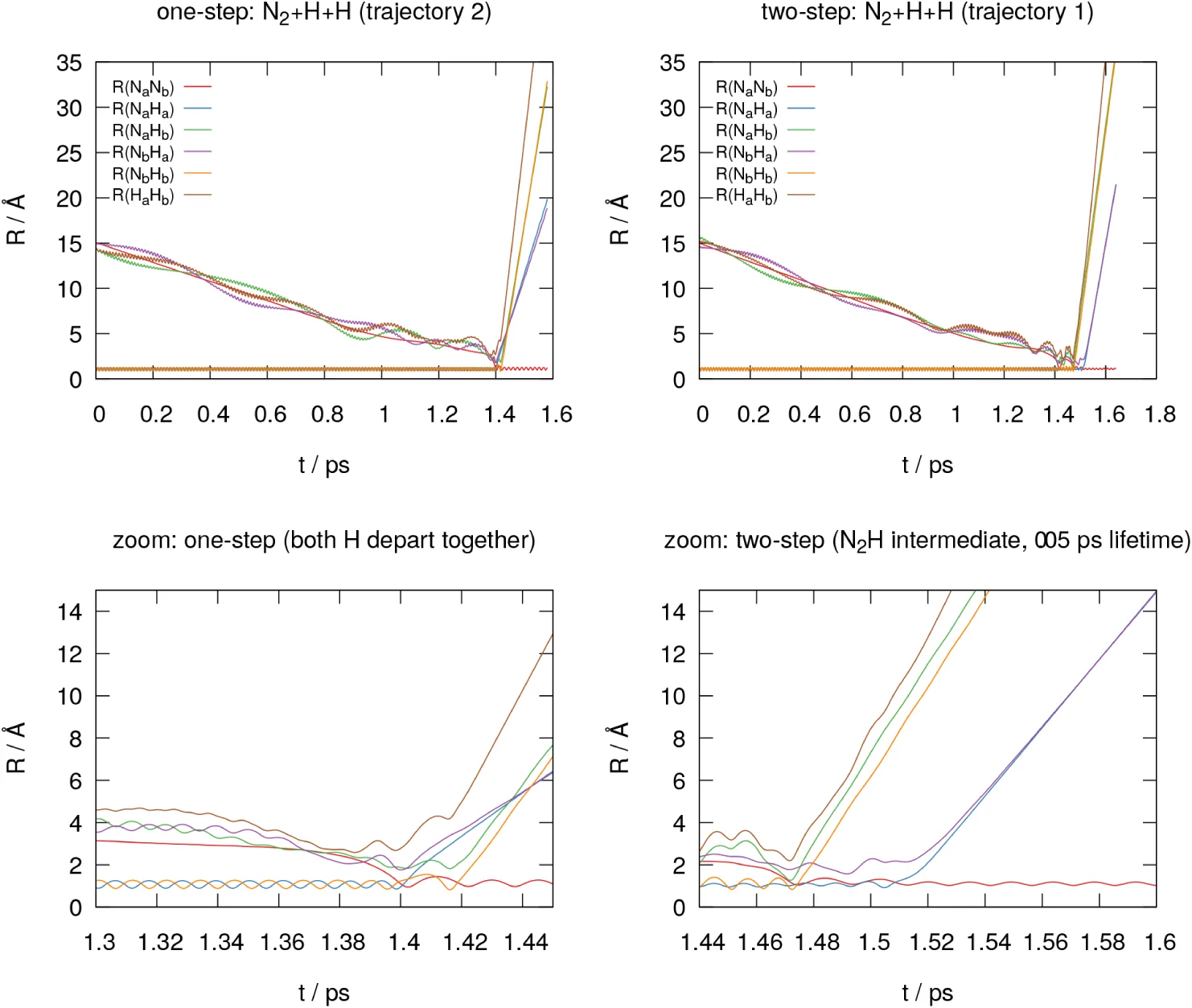
Interatomic distances vs time for representative one-step (left) and two-step (right) reactive trajectories, NH­(1, 1) + NH­(1, 1) → N_2_ + H + H, *E*_tr_ = 1.0 kcal mol^–1^. Bottom row: close-up of the departure region.

Table 1 collects the one-step/two-step branching fraction for all ten (*v*, *v*′) combinations at selected translational energies (see Figure 5 for the full *E*_tr_ dependence). For the asymmetric combinations NH­(*v*, 1) + NH­(0, 1), the one-step fraction decreases gradually from ∼70–75% for *v* = 1, close to the value reported for the rotational ground state, to ∼57–61% for *v* = 5, with only a weak dependence on *E*_tr_. The combinations in which both reactants are excited, NH­(*v*, 1) + NH­(*v*, 1) with *v* ≥ 2, show a different *E*_tr_ dependence: the one-step fraction drops at low translational energy, to as low as 43.6% for (2, 2) at *E*_tr_ = 0.1 kcal mol^–1^, and rises to ∼62–70% at *E*_tr_ ≥ 5 kcal mol^–1^. At *E*_tr_ = 10 kcal mol^–1^ the one-step fraction of the (*v*, *v*) states with *v* ≥ 3 is in fact *larger* than that of the corresponding (*v*, 0) states (e.g., 67.6% vs 57.1% for (5, 5) and (5, 0)).

**Table 1:** One-Step and Two-Step Mechanism Percentages (of Channel-12 Reactive Trajectories) for NH­(*v*, 1) + NH­(*v*′, 1) → N_2_ + H + H, as a Function of *E*_tr_ (kcal mol^–1^)­^a^

		*E*_tr_ = 0.1		*E*_tr_ = 1		*E*_tr_ = 5		*E*_tr_ = 10	
*v*	*v*′	1-step	2-step	1-step	2-step	1-step	2-step	1-step	2-step
1	0	73.8 ± 2.7	26.2	73.3 ± 1.0	26.7	70.0 ± 1.0	30.0	69.3 ± 1.0	30.7
1	1	75.2 ± 2.4	24.8	72.8 ± 1.0	27.2	73.8 ± 0.9	26.2	72.8 ± 0.9	27.2
2	0	73.6 ± 2.8	26.4	69.8 ± 1.1	30.2	68.5 ± 1.0	31.5	65.4 ± 1.0	34.6
2	2	43.6 ± 2.5	56.4	60.2 ± 1.0	39.8	66.9 ± 1.0	33.1	69.7 ± 0.9	30.3
3	0	68.5 ± 2.7	31.5	64.5 ± 1.1	35.5	63.2 ± 1.0	36.8	63.1 ± 1.0	36.9
3	3	47.6 ± 2.3	52.4	53.7 ± 1.0	46.3	62.2 ± 1.0	37.8	66.2 ± 1.0	33.8
4	0	61.3 ± 2.9	38.7	60.1 ± 1.1	39.9	58.1 ± 1.0	41.9	59.3 ± 1.0	40.7
4	4	55.6 ± 2.4	44.4	54.8 ± 1.0	45.2	60.9 ± 1.0	39.1	65.9 ± 1.0	34.1
5	0	61.0 ± 3.0	39.0	59.7 ± 1.1	40.3	58.4 ± 1.0	41.6	57.1 ± 1.0	42.9
5	5	52.9 ± 2.4	47.1	56.6 ± 1.0	43.4	61.6 ± 0.9	38.4	67.6 ± 0.9	32.4

a The one-step percentages are given with their binomial standard uncertainty, [*p*(1 – *p*)/*N*_12_]^1/2^; the two-step percentage is its complement and carries the same uncertainty.

**Figure 5:**
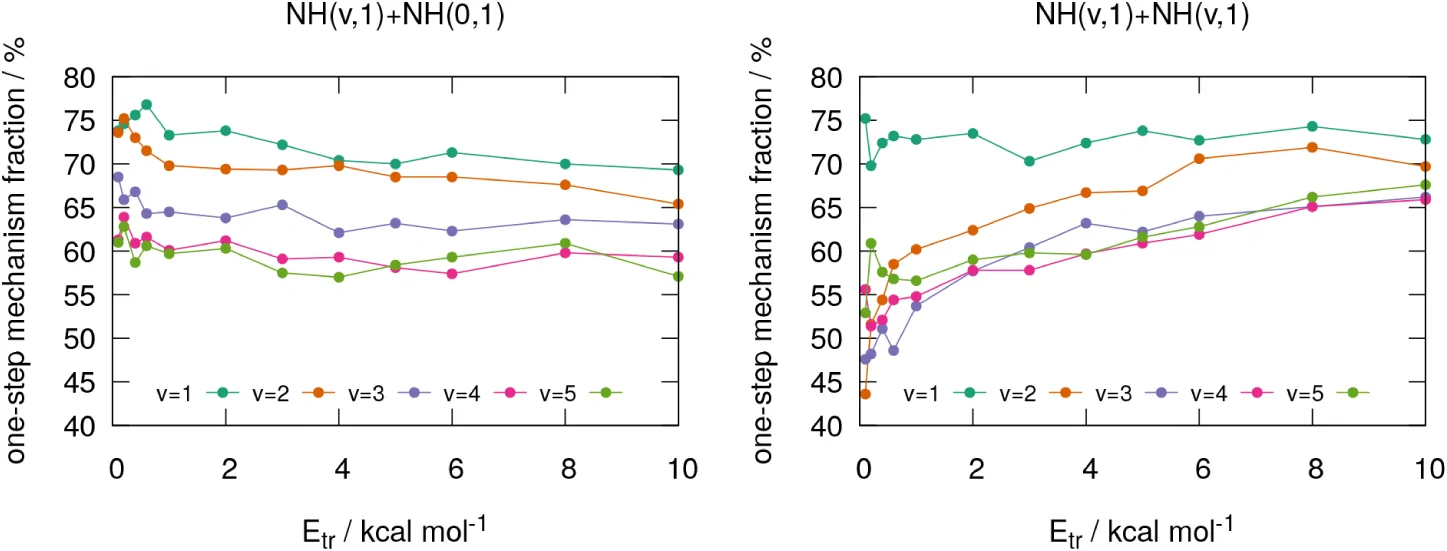
One-step mechanism fraction (% of channel-12 reactive trajectories) vs *E*_tr_, for the asymmetric NH­(*v*, 1) + NH­(0, 1) (left) and symmetric NH­(*v*, 1) + NH­(*v*, 1) (right) combinations, *v* = 1–5.

Because (*v*, *v*) and (*v*, 0) differ in total vibrational energy as well as in its distribution, these comparisons alone do not separate the two effects. Two pairs in the present data set have nearly equal total vibrational excitation (Table 10). For (1, 1) and (2, 0) (17.9 vs 17.4 kcal mol^–1^), the one-step fractions agree within their statistical uncertainties at the lowest collision energies (75.2 ± 2.4% vs 73.6 ± 2.8% at *E*_tr_ = 0.1 kcal mol^–1^), but for *E*_tr_ ≥ 5 kcal mol^–1^ the (1, 1) state has a significantly larger one-step fraction (72.8 ± 0.9% vs 65.4 ± 1.0% at 10 kcal mol^–1^). For (2, 2) and (4, 0) (34.8 vs 33.1 kcal mol^–1^), the one-step fraction of (2, 2) is much smaller at low collision energy (43.6 ± 2.5% vs 61.3 ± 2.9% at *E*_tr_ = 0.1 kcal mol^–1^, about 4.6 standard deviations, and 51.6 ± 1.6% vs 63.9 ± 1.8% at 0.2 kcal mol^–1^), the two coincide at 1.0 kcal mol^–1^ (60.2% vs 60.1%), and the order is reversed for *E*_tr_ ≥ 3 kcal mol^–1^ (69.7 ± 0.9% vs 59.3 ± 1.0% at 10 kcal mol^–1^). The mixed state (3, 1), which carries nearly the same total excitation (34.4 kcal mol^–1^), does not interpolate between the two: its one-step fraction is almost independent of *E*_tr_ (77.7 ± 2.7% at 0.1, 72.3 ± 1.1% at 1.0 and 70.6 ± 1.0% at 10 kcal mol^–1^), close to that of (1, 1). It is larger than that of (4, 0) at every *E*_tr_ (by 3.5–10 standard deviations), larger than that of (2, 2) up to 4 kcal mol^–1^, and equal to it within the statistical uncertainty for *E*_tr_ ≥ 5 kcal mol^–1^. Its reactive cross section lies between those of (4, 0) and (2, 2) for *E*_tr_ ≥ 0.2 kcal mol^–1^ (at 0.1 kcal mol^–1^ the three agree within their uncertainties). At nearly matched total energy, then, the distribution of the vibrational energy between the reactants does affect the branching, but not monotonically. At high *E*_tr_, sharing the excitation between both reactants, whether partially (3, 1) or equally (2, 2), favors the one-step mechanism relative to (4, 0). At low *E*_tr_, the ordering is (3, 1) >; (4, 0) >; (2, 2), so the enhanced two-step (N_2_H-intermediate) contribution is specific to (2, 2), in which both reactants carry at least two vibrational quanta, rather than a general consequence of sharing the excitation. One plausible interpretation is that at low collision energy the complex formed by two strongly excited reactants lives longer and has more time to reach the N_2_H intermediate before dissociating. These conclusions refer to the singlet surface. The original study, which examined only *v*, *v*′ = 0, 1, could not have seen this effect.

Applying the IVEQMT threshold introduced below (in the Reactive Cross Section subsection) to the mechanism classification changes the one-step percentage by only 0.09 percentage points on average, and by at most 0.71 points across all 120 (*v*, *v*′, *E*_tr_) combinations, as shown for a representative subset in Table 2 and, over the full *E*_tr_ range, in Figure 6. The mechanism branching discussed above is therefore essentially unaffected by ZPE leakage.

**Table 2:** One-Step Mechanism Percentage, Raw QCT vs IVEQMT-Corrected, for a Representative Subset of Combinations (cf. Table 1 for the Full Raw-QCT Grid)

*v*, *v*′	*E*_tr_	1-step % (QCT)	1-step % (IVEQMT)
(1, 0)	0.1	73.8	73.7
(1, 0)	1.0	73.3	73.6
(1, 0)	5.0	70.0	70.4
(1, 0)	10.0	69.3	69.1
(1, 1)	0.1	75.2	75.4
(1, 1)	1.0	72.8	72.9
(1, 1)	5.0	73.8	73.9
(1, 1)	10.0	72.8	73.0
(4, 0)	0.1	61.3	61.6
(4, 0)	1.0	60.1	60.1
(4, 0)	5.0	58.1	58.1
(4, 0)	10.0	59.3	59.3
(4, 4)	0.1	55.6	55.6
(4, 4)	1.0	54.8	54.8
(4, 4)	5.0	60.9	60.9
(4, 4)	10.0	65.9	65.8
(2, 2)	0.1	43.6	43.6
(2, 2)	1.0	60.2	60.3
(2, 2)	5.0	66.9	67.0
(2, 2)	10.0	69.7	69.8
(5, 5)	0.1	52.9	52.9
(5, 5)	1.0	56.6	56.6
(5, 5)	5.0	61.6	61.7
(5, 5)	10.0	67.6	67.5

**Figure 6:**
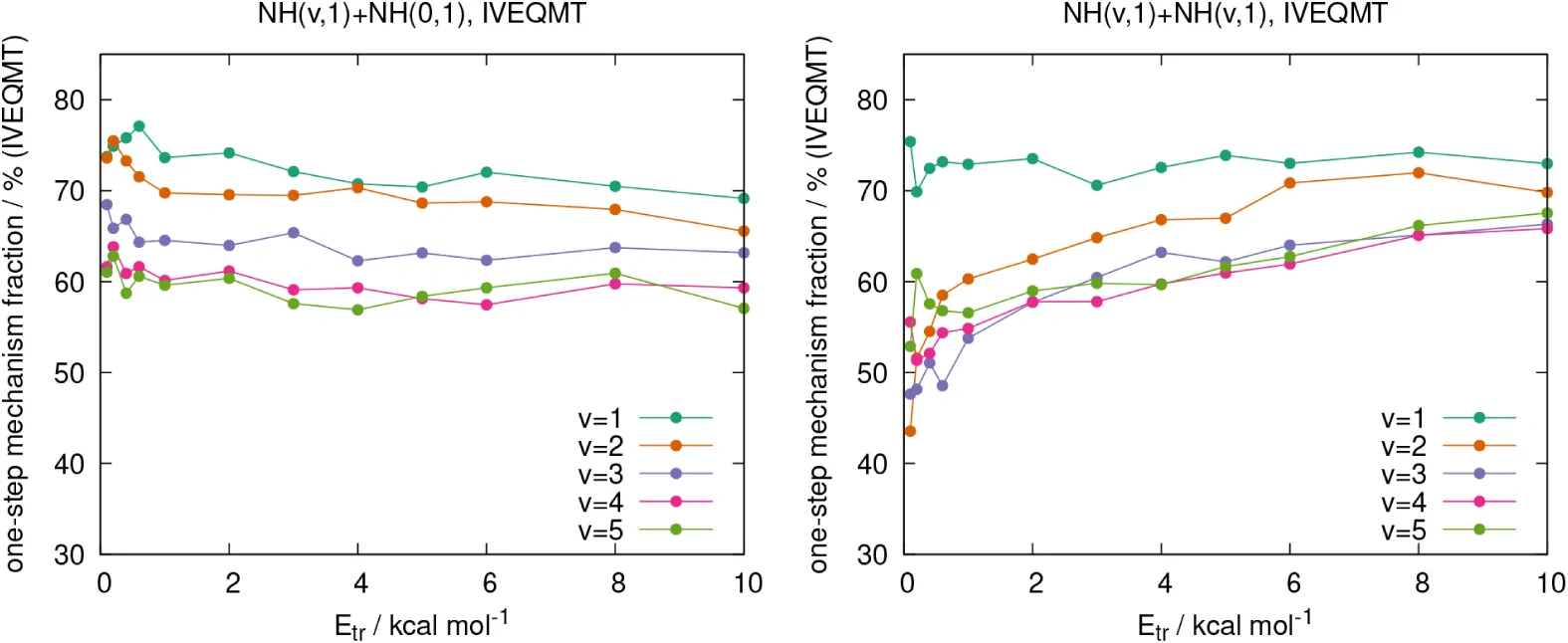
Same as Figure 5, but with the IVEQMT correction applied to the mechanism classification.

### Reactive Cross Section

Table 3 presents the channel-12 reactive cross section, σ_12_ = π*b*_max_^2^ *N*_12_/*N*_T_, for all ten (*v*, *v*′) combinations. As in the rotational study, σ_12_(*E*_tr_) shows a pronounced maximum around *E*_tr_ ≈ 0.6 – 1.0 kcal mol^–1^ for every combination. This is consistent with the long-range-dominated capture/reorientation mechanism discussed in ref 11: at low *E*_tr_ the reactants have time to reorient toward the attractive collinear approach, but this orientation becomes repulsive at short-range; at higher *E*_tr_, trajectories retain more of the barrier-free short-range capture character of the perpendicular approach.

**Table 3:** Reactive Cross Section σ_12_ (in Å^2^) for NH­(*v*, 1) + NH­(*v*′, 1) → N_2_ + H + H as a Function of Translational Energy *E*_tr_ (kcal mol^–1^)­^a^

*E*_tr_	(1, 0)	(1, 1)	(2, 0)	(2, 2)	(3, 0)	(3, 3)	(4, 0)	(4, 4)	(5, 0)	(5, 5)
0.1	6.87	6.43	6.16	7.02	6.76	8.40	6.75	8.66	6.23	9.60
0.2	14.30	16.35	14.31	17.02	13.86	17.93	14.20	17.57	12.67	17.42
0.4	25.61	28.03	24.76	28.18	25.09	28.19	24.08	27.71	22.40	27.80
0.6	28.68	31.94	29.23	34.67	29.58	34.13	28.57	32.35	27.54	31.56
1.0	31.34	34.47	30.78	34.80	30.65	34.63	30.35	33.72	29.19	32.75
2.0	28.43	30.91	28.51	32.49	28.94	32.10	27.63	31.55	26.96	30.60
3.0	25.02	26.28	24.85	27.78	24.74	28.72	24.94	28.82	24.59	28.57
4.0	22.73	24.25	23.11	26.41	24.12	26.97	23.83	27.06	23.19	26.97
5.0	22.45	23.68	21.99	24.71	22.61	25.84	22.79	26.14	22.53	26.67
6.0	21.60	22.47	21.83	24.97	22.05	24.64	22.21	25.34	22.34	25.29
8.0	20.87	21.75	20.91	23.10	21.05	23.79	21.46	23.87	20.99	23.97
10.0	20.06	20.46	19.66	22.04	20.29	22.48	20.48	22.59	20.27	22.30

a *N_T_* = 4000 for every entry.

The symmetric combinations show a consistent, modest enhancement of σ_12_ relative to the asymmetric ones at the same *v* and *E*_tr_: at *E*_tr_ = 1.0 kcal mol^–1^, for example, σ_12_ increases from 31.3 Å^2^ for (1, 0) to 34.5 Å^2^ for (1, 1), and from 30.4 Å^2^ for (4, 0) to 33.7 Å^2^ for (4, 4)—roughly a 10% enhancement when both reactants, rather than one, carry the vibrational excitation. The effect is much smaller than the one previously reported for rotational excitation, in line with the original study’s expectation that vibrational energy affects the reactivity of this system comparatively little.

To test how sensitive the raw QCT statistics above are to ZPE leakage, we repeated the channel-12 trajectory count after applying the intermediate vibrational-energy quantum-mechanical threshold (IVEQMT) correction for ZPE leakage, in the variant recently used for a chemically related system by our group:^20,41^ a reactive trajectory is discarded if the vibrational energy of the product N_2_ falls below one-half of its ZPE (*E*_th_ = 3.36/2 = 1.68 kcal mol^–1^, using ω_*e*_ = 2358.57 cm^–1^ and ω_*e*_ *x*_*e*_ = 14.32 cm^–1^ for N_2_). Table 4 shows the resulting cross sections for a representative subset of combinations, and Figure 7 presents the corresponding QCT and IVEQMT excitation functions over the full translational-energy range, with error bars computed as in eq 2 of ref 11, in the same style used for the analogous comparison in the N_2_ + H_2_ IVEQMT study.^20^ The correction reduces σ_12_ by 0.6% on average (up to 3.5% for a single combination), and the QCT and IVEQMT curves are indistinguishable within their error bars across the whole range and for every combination shown. As for the stricter VEQMT criterion applied to the rotational-energy data set in ref 11 (where essentially no channel-12 trajectories were rejected), ZPE leakage therefore has a negligible impact on the channel-12 statistics, including for the vibrationally excited states explored here.

**Table 4:** Reactive Cross Section σ_12_ (Å^2^), Raw QCT vs IVEQMT-corrected^41^ (1/2-ZPE Threshold on Product N_2_ Vibrational Energy, *E*_th_ = 1.68 kcal mol^–1^), for Representative Combinations

*v*, *v*′	*E*_tr_	σ_12_^QCT^	σ_12_^IVEQMT^	Reduction (%)
(1, 0)	1.0	31.34	30.76	1.86
(1, 0)	5.0	22.45	21.95	2.27
(1, 1)	1.0	34.46	33.86	1.75
(1, 1)	5.0	23.68	23.27	1.72
(2, 0)	1.0	30.78	30.50	0.92
(2, 0)	5.0	21.99	21.71	1.25
(2, 2)	1.0	34.80	34.69	0.32
(2, 2)	5.0	24.71	24.59	0.49
(3, 0)	1.0	30.65	30.54	0.36
(3, 0)	5.0	22.61	22.46	0.68
(3, 3)	1.0	34.63	34.59	0.13
(3, 3)	5.0	25.84	25.80	0.16
(4, 0)	1.0	30.35	30.32	0.10
(4, 0)	5.0	22.79	22.68	0.49
(4, 4)	1.0	33.72	33.72	0.00
(4, 4)	5.0	26.14	26.14	0.00
(5, 0)	1.0	29.19	29.12	0.25
(5, 0)	5.0	22.53	22.47	0.23
(5, 5)	1.0	32.75	32.73	0.04
(5, 5)	5.0	26.67	26.65	0.08

**Figure 7:**
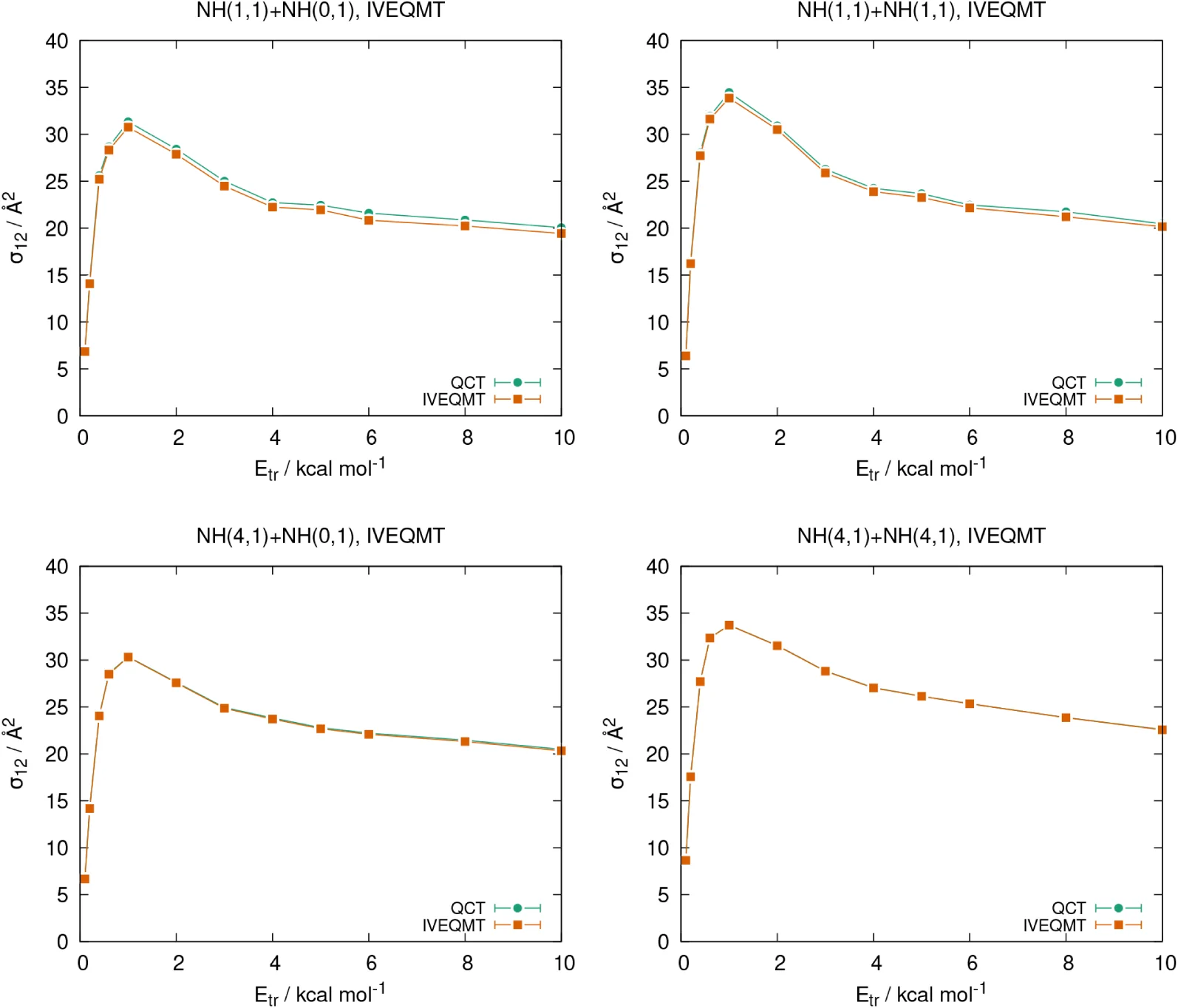
Excitation functions σ_12_ vs *E*_tr_, raw QCT compared with IVEQMT-corrected, with error bars, for four representative NH­(*v*, 1) + NH­(*v*′, 1) combinations.

Table 5 reports the IVEQMT-corrected cross section for the complete (*v*, *v*′) × *E*_tr_ grid (cf. Table 3 for the raw QCT values), and Figure 8 shows the corresponding excitation functions for all ten combinations, directly comparable to Figure 9 below.

**Table 5:** IVEQMT-Corrected Reactive Cross Section σ_12_ (in Å^2^) for NH­(*v*, 1) + NH­(*v*′, 1) → N_2_ + H + H as a Function of Translational Energy *E*_tr_ (kcal mol^–1^); cf. Table 3 for the Raw QCT Values

*E*_tr_	(1, 0)	(1, 1)	(2, 0)	(2, 2)	(3, 0)	(3, 3)	(4, 0)	(4, 4)	(5, 0)	(5, 5)
0.1	6.84	6.39	6.16	7.02	6.76	8.40	6.67	8.66	6.23	9.60
0.2	14.08	16.22	14.25	17.02	13.86	17.21	14.18	17.56	12.67	17.42
0.4	25.21	27.72	24.54	28.07	25.01	28.19	24.05	27.71	22.40	27.80
0.6	28.33	31.63	29.01	34.60	29.48	34.13	28.49	32.34	27.51	31.56
1	30.76	33.86	30.50	34.69	30.54	34.59	30.32	33.72	29.12	32.73
2	27.88	30.51	28.19	32.31	28.74	32.07	27.57	31.53	26.88	30.60
3	24.49	25.89	24.50	27.68	24.60	28.69	24.86	28.81	24.54	28.54
4	22.24	23.89	22.78	26.27	23.89	26.95	23.71	27.03	23.15	26.94
5	21.95	23.27	21.71	24.59	22.46	25.80	22.68	26.14	22.47	26.65
6	20.84	22.16	21.45	24.81	21.90	24.60	22.09	25.35	22.30	25.28
8	20.24	21.21	20.50	22.90	20.88	23.77	21.32	23.86	20.91	23.96
10	19.43	20.16	19.24	21.92	20.04	22.40	20.33	22.56	20.16	22.27

**Figure 8:**
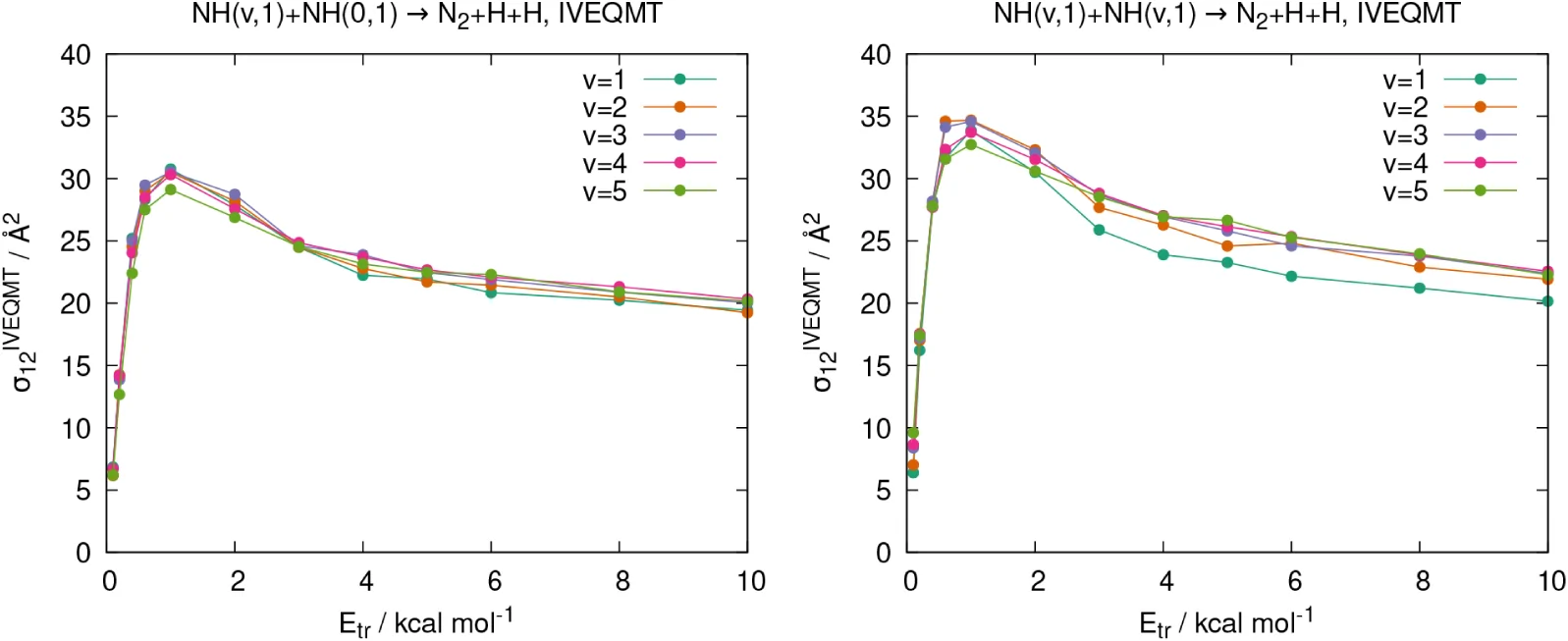
Excitation functions σ_12_^IVEQMT^ vs *E*_tr_, for all ten combinations, split into the asymmetric NH­(*v*, 1) + NH­(0, 1) (left) and symmetric NH­(*v*, 1) + NH­(*v*, 1) (right) sets; cf. the raw QCT equivalent in Figure 9 below.

**Figure 9:**
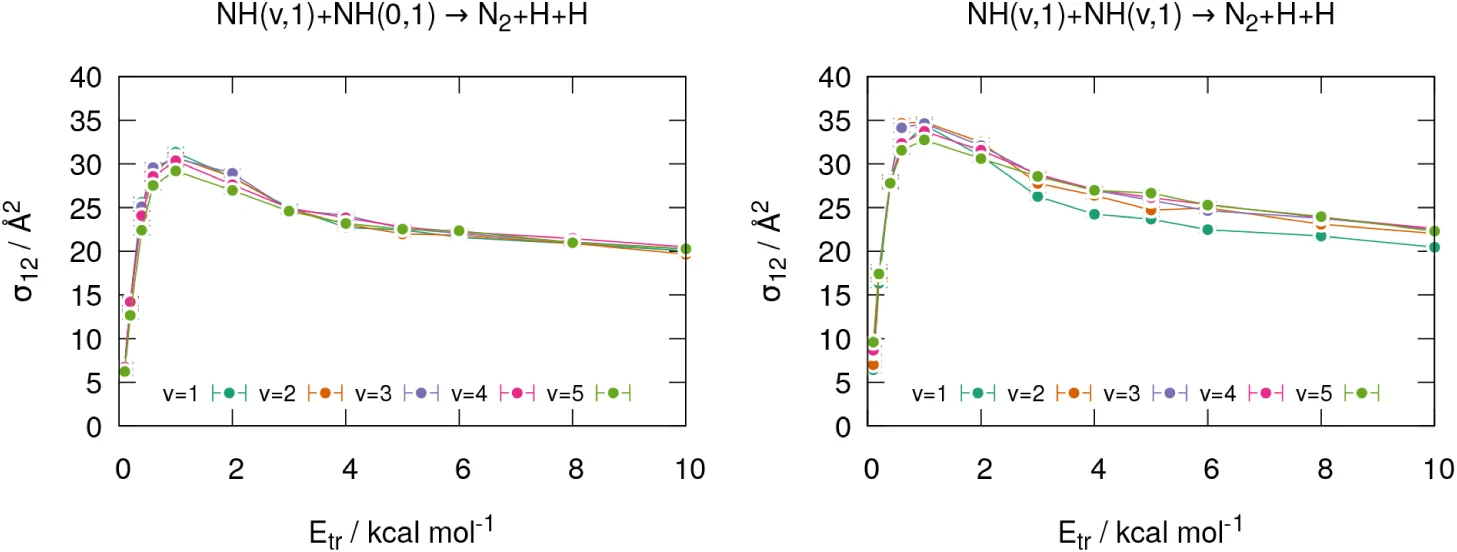
Reactive cross section σ_12_ vs *E*_tr_, for the asymmetric NH­(*v*, 1) + NH­(0, 1) (left) and symmetric NH­(*v*, 1) + NH­(*v*, 1) (right) combinations, *v* = 1–5. Error bars follow eq 2 of ref 11.

### Excitation Functions

Figure 9 shows the excitation functions for all ten combinations, split into the asymmetric and symmetric sets in analogy with Figures 7 and Figure 8 of ref 11, which made the corresponding comparison for rotational excitation. The characteristic low-energy suppression and the maximum near 1 kcal mol^–1^ hold across the whole *v* = 1–5 range, with the curves for different *v* staying close to one another. Vibrational excitation, unlike rotational excitation, therefore does not change the reactivity pattern of this system qualitatively; it only modulates it to the modest extent discussed above.

### Vibrational, Rotational, and Translational Energy Partitioning of Nascent N_2_

Figure 10 shows the vibrational energy distribution of the nascent N_2_ product, separated by microscopic mechanism, for representative combinations at *E*_tr_ = 1.0 kcal mol^–1^. As in the rotational study, the one-step mechanism produces N_2_ with higher vibrational energy content than the two-step mechanism, for every combination examined (Table 6). The two-step pathway allows at least one full N–N oscillation during the ∼0.05 ps lifetime of the N_2_H intermediate, which redistributes vibrational energy before the second hydrogen departs.

**Table 6:** Mean Vibrational, Rotational, and Translational Energy (kcal mol^–1^) of Nascent N_2_, and of the Relative N_2_···H Translational Motion, Produced via the One-Step and Two-Step Mechanisms, for Selected NH­(*v*, 1) + NH­(*v*′, 1) Combinations at *E*_tr_ = 1.0 and 5.0 kcal mol^a^ ^–1^

		one-step			two-step			
*v*, *v*′	*E*_tr_	⟨*E*_vib_⟩	⟨*E*_rot_⟩	⟨*E*_trans_⟩	⟨*E*_vib_⟩	⟨*E*_rot_⟩	⟨*E*_trans_⟩	*N*_1_/*N*_2_
(1, 0)	1.0	34.0	5.8	20.6	25.2	5.9	25.1	1383/419
(1, 0)	5.0	27.6	12.7	22.0	21.2	11.7	25.7	1544/537
(1, 1)	1.0	38.5	6.1	23.6	30.7	5.8	27.4	1578/526
(1, 1)	5.0	35.5	13.2	23.7	28.4	11.4	27.1	1716/512
(4, 0)	1.0	54.2	5.1	20.6	42.2	5.2	27.7	1200/733
(4, 0)	5.0	46.8	13.4	21.7	39.0	12.9	26.7	1301/838
(4, 4)	1.0	69.3	4.4	32.9	62.7	4.8	35.6	1335/1062
(4, 4)	5.0	61.4	15.6	33.4	60.1	12.7	36.0	1565/949

a Only trajectories that end with a free N_2_ fragment are included; the 3.5% of channel-12 trajectories that donot (see calculation details) are excluded. *N*_1_/*N*_2_ is the number of one-step/two-step trajectories averaged.

**Figure 10:**
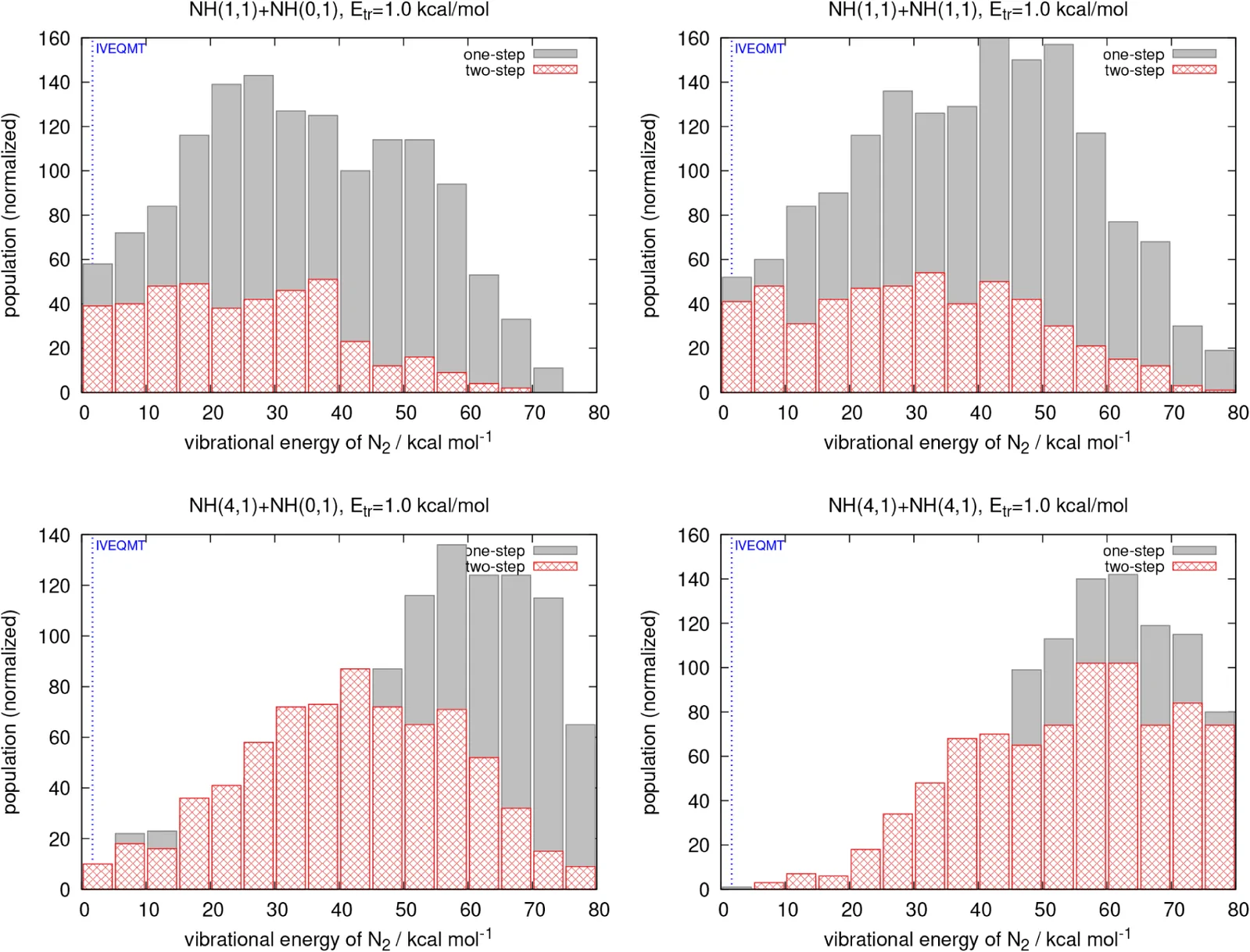
Vibrational energy distribution of nascent N_2_, separated by mechanism, for selected NH­(*v*, 1) + NH­(*v*′, 1) combinations at *E*_tr_ = 1.0 kcal mol^–1^. The dotted vertical line marks the IVEQMT threshold (*E*_th_ = 1.68 kcal mol^–1^); only a few trajectories in the leftmost bin fall below it.

Increasing the reactants’ vibrational excitation shifts the product N_2_ vibrational energy distribution upward: the mean one-step vibrational energy rises from 34.0 kcal mol^–1^ for (1, 0) to 54.2 kcal mol^–1^ for (4, 0), and from 38.5 kcal mol^–1^ for (1, 1) to 69.3 kcal mol^–1^ for (4, 4), at *E*_tr_ = 1.0 kcal mol^–1^. Reactant vibrational energy is thus efficiently funneled into the product’s vibrational degree of freedom, rather than being redistributed into rotation or translation; this effect is somewhat more pronounced for the symmetric (both-excited) combinations. Reactant vibrational excitation controls product energy disposal in much the same way in the chemically related N_2_ + H_2_ system, as recently reported by our group, both in reactive collisions at high translational and vibrational energies^20^ and in the corresponding nonreactive vibrational relaxation channel.^21^ Efficient vibrational-energy funneling may therefore be general to four-atom N–H–H–N/N–N–H–H exchange dynamics on barrierless or near-barrierless potential energy surfaces.

Table 7 repeats this analysis for the subset of trajectories that pass the IVEQMT threshold. As Figure 10 suggests, the effect is small: removing the few subthreshold trajectories shifts the mean one-step vibrational energy upward by at most 0.7 kcal mol^–1^ (e.g., 34.0→34.5 kcal mol^–1^ for (1, 0) at *E*_tr_ = 1.0 kcal mol^–1^) and the two-step value by at most 0.9 kcal mol^–1^, with the rotational and translational columns essentially unchanged, matching the negligible cross-section and rate-constant corrections found elsewhere in this work.

**Table 7:** Same as Table 6, but Restricted to Trajectories Passing the IVEQMT Threshold (*E*_vib_(N_2_) > 1.68 kcal mol^–1^)­^a^

		one-step (IVEQMT)			two-step (IVEQMT)			
*v*, *v*′	*E*_tr_	⟨*E*_vib_⟩	⟨*E*_rot_⟩	⟨*E*_trans_⟩	⟨*E*_vib_⟩	⟨*E*_rot_⟩	⟨*E*_trans_⟩	*N*_1_/*N*_2_
(1, 0)	1.0	34.5	5.8	20.4	26.1	5.8	24.7	1363/404
(1, 0)	5.0	28.0	12.7	21.8	22.1	11.7	25.1	1518/513
(1, 1)	1.0	39.2	6.0	23.3	31.4	5.8	27.0	1552/514
(1, 1)	5.0	36.1	13.2	23.5	29.1	11.4	26.7	1689/499
(4, 0)	1.0	54.2	5.1	20.6	42.3	5.2	27.7	1199/732
(4, 0)	5.0	47.0	13.4	21.6	39.2	12.9	26.5	1295/833
(4, 4)	1.0	69.3	4.4	32.9	62.7	4.8	35.6	1335/1062
(4, 4)	5.0	61.4	15.6	33.4	60.1	12.7	36.0	1565/949

a *N*_1_/*N*_2_ here counts only trajectories surviving the threshold.

Table 6 also collects the mean rotational energy of the nascent N_2_ and the mean relative translational energy between the N_2_ and one departing H atom, extracted from the same trajectory ensembles using the final-state energy analysis internally recorded by the trajectory code for each reactive event. Two trends parallel those reported for the rotational ground state in ref 11. First, the product rotational energy increases markedly with translational energy at fixed (*v*, *v*′), from 5.8 to 12.7 kcal mol^–1^ for (1, 0) and from 4.4 to 15.6 kcal mol^–1^ for (4, 4) as *E*_tr_ is raised from 1.0 to 5.0 kcal mol^–1^. The efficient translational-to-rotational energy transfer identified previously therefore persists across the whole range of reactant vibrational excitation explored here. Second, the two-step mechanism consistently channels more energy into product translation than the one-step mechanism, for every combination and at both translational energies examined (e.g., one-step vs two-step: 20.6 vs 25.1 kcal mol^–1^ for (1, 0) at *E*_tr_ = 1.0 kcal mol^–1^; 32.9 vs 35.6 kcal mol^–1^ for (4, 4)). The pattern reported for the rotational ground state therefore holds for vibrationally excited reactants too, and is consistent with the two-step complex’s longer lifetime, which allows more of the reaction exothermicity to flow into the relative motion of the separating fragments before the final hydrogen departs. Unlike the vibrational energy content, the product’s rotational energy is comparable between the two mechanisms and does not show as clear a systematic trend with reactant vibrational excitation. Reactant vibrational energy therefore goes preferentially into product vibration, and any residual effect on the departure dynamics appears in product translation rather than in product rotation.

As a check on these extracted quantities, Table 8 compares, for the one-step mechanism, the total energy nominally available to the products with the sum of the extracted N_2_ vibrational, rotational, and N_2_–H relative translational energies. The available energy is *E*_tr_ plus the reactants’ initial vibrational and rotational energies (read directly from the trajectory input, using the rigid-rotor value *E*_rot_(*j* = 1) = *B*_*e*_ *j*(*j* + 1) = 0.0955 kcal mol^–1^ per NH with *B*_*e*_ = 16.70 cm^–1^), plus the N_2_ + H + H/NH + NH electronic asymptote gap of 65.02 kcal mol^–1^ (from the potential energy values referenced to the N_2_ + H_2_ dissociation limit, consistent with Table 1 of ref 11.

**Table 8:** Partial Energy-Balance Check for the One-Step Mechanism: Total Energy Available to the Products (*E*_tr_ + Reactant Vibrational + Rotational Energy + the N_2_ + H + H/NH + NH Electronic Asymptote Gap, 65.02 kcal mol^–1^) vs the Sum of the Mean N_2_ Vibrational, Rotational and N_2_–H Relative Translational Energies Extracted from the Trajectories (Table 6)­^a^

*v*, *v*′	*E*_tr_	*E*_available_	*E*_vib_ + *E*_rot_ + *E*_trans_(N_2_–H)	gap	gap (%)
(1, 0)	1	84.4	60.4	23.9	28.4
(1, 0)	5	88.4	62.3	26.1	29.5
(1, 1)	1	93.2	68.2	25.0	26.8
(1, 1)	5	97.2	72.4	24.8	25.5
(4, 0)	1	108.3	80.0	28.3	26.1
(4, 0)	5	112.3	81.9	30.3	27.0
(4, 4)	1	141.0	106.7	34.4	24.4
(4, 4)	5	145.0	110.4	34.6	23.9

a The gap is attributed to the kinetic energy of the second, untracked H atom in the trajectory code’s two-fragment final-state analysis (see text).

We find a systematic difference of 24–30% between the available energy and the sum of the extracted N_2_ vibrational, rotational, and N_2_–H translational energies in every combination checked for the one-step mechanism. This difference corresponds to the kinetic energy carried by the second departing hydrogen atom (much lighter than the N_2_ fragment, and hence disproportionately fast in an impulsive breakup), which is not reported by the trajectory code’s standard two-fragment final-state analysis—routinely used to characterize the pseudodiatomic product pair, here N_2_ against one H atom. This is not a methodological error or a sign of energy nonconservation: it is simply the kinetic energy of the third fragment, not isolated by the code’s two-fragment output but recoverable from the total energy balance, as done here. We confirmed this directly by computing, for every individual reactive trajectory, the residual *E*_available_ – (*E*_vib_ + *E*_rot_ + *E*_trans_) as a proxy for the second-H kinetic energy. Across all 87 444 channel-12 trajectories of the (1, 0), (1, 1), (4, 0) and (4, 4) combinations, over the full *E*_tr_ range, this residual is negative in only 8 cases, the largest at −3.1 kcal mol^–1^, consistent with the small fraction of trajectories whose energy conservation is worse than 1% (Figure 3); a genuine energy-balance error would instead produce positive and negative residuals with comparable frequency. The resulting per-trajectory distributions, shown in Figure 11 for four representative combinations, are broad and unimodal, shift to higher energies with increasing reactant vibrational excitation, and for (4, 4) reach about 100 kcal mol^–1^, with a small tail (0.2% of the trajectories) beyond it: direct evidence that the title reaction is a source of kinetically hot H atoms. The mean of this residual is also systematically larger for the two-step mechanism than for the one-step mechanism at fixed (*v*, *v*′) and *E*_tr_ (e.g., one-step vs two-step: 28.4% vs 33.3% of the available energy for (*v*, *v*′) = (1, 0) at *E*_tr_ = 1.0 kcal mol^–1^), reflecting a mechanism-dependent difference in how the two departing hydrogen atoms share the available energy.

**Figure 11:**
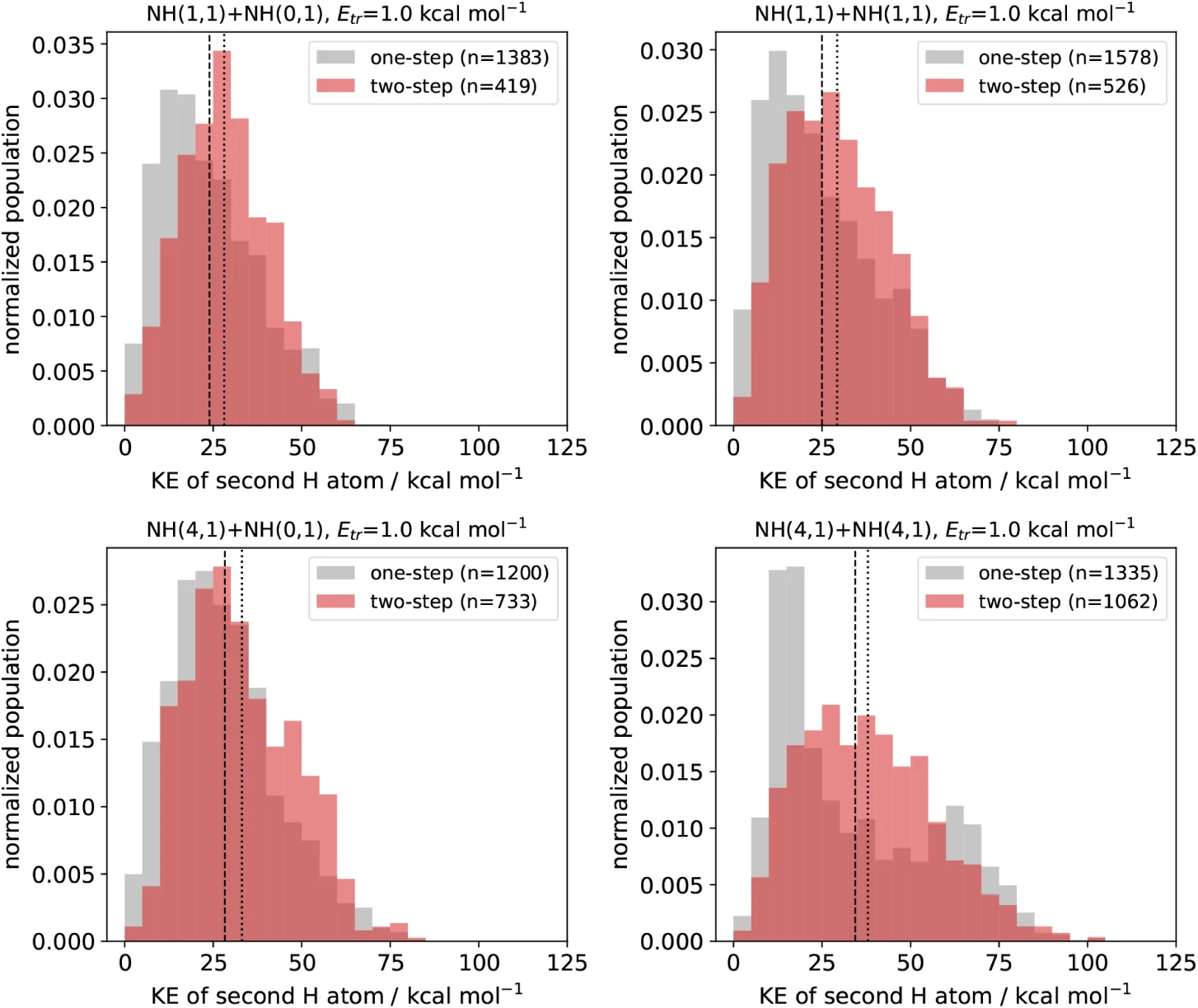
Kinetic-energy distribution of the second, otherwise untracked H atom (obtained from the per-trajectory total-energy balance), separated by mechanism, for four representative (*v, v*′) combinations at *E*_tr_ = 1.0 kcal mol^–1^.

### Rate Constants

Initial-state-specific singlet-surface rate constants, *k*(*T*; *v*, *v*′), were obtained by numerically integrating σ_12_(*E*_tr_) over the available translational-energy grid according to eq 3 of ref 11 ^,^ which includes the electronic-degeneracy factor *g*_*e*_(*T*) = 1/9 appropriate to a singlet N_2_H_2_ product formed from two triplet NH radicals.^42,43^ Because only the singlet surface is included, these are the singlet contributions to the NH + NH → N_2_ + H + H rate constant, not total rate constants. We fitted the results to the same modified Arrhenius–Kooij form, *k*(*T*) = *AT^n^* (1 – *dE*_a_/*T*)^1/*d*^, proposed by Aquilanti and co-workers^44,45^ and used in our previous study. Table 9 reports the fitted parameters obtained both from the raw QCT σ_12_(*E*_tr_) and from the IVEQMT-corrected excitation functions discussed above (Figure 7), mirroring the side-by-side QCT/VEQMT presentation in Table 5 of ref 11. Given the small cross-section corrections found above, the two sets of rate constants agree closely: Figure 12 compares the resulting *k*(*T*) curves for two representative combinations, and the difference stays below 3% across the whole temperature range explored (200–3000 K) for every combination checked in Table 10 (Figure 13).

**Table 9:** Fitted Parameters for *k*(*t*) =*AT* ^*n*^(1 – *dE*_a_/*T*)^1/*d*^ (in 10^–11^ cm^3^ s^–1^) for NH­(*v*, 1) + NH­(*v*′, 1) → N_2_ + H + H Singlet-Surface Rate Constants, Comparing Raw QCT and IVEQMT-Corrected σ_12_(*E*_tr_) Integrated over 0.1 ≤ *E*_tr_/kcal mol^–1^ ≤ 10 (See Caveat in Text on the High-*T* Range)

	QCT				IVEQMT			
*v*, *v*′	*A*	*n*	*E*_*a*_ (K)	*d*	*A*	*n*	*E*_*a*_ (K)	*d*
(1, 0)	6.97 × 10^3^	–0.867	1969.3	–0.514	4.73 × 10^5^	–1.332	4283.2	–0.451
(1, 1)	6.02 × 10^4^	–1.101	2979.7	–0.493	5.02 × 10^5^	–1.333	4255.1	–0.456
(2, 0)	5.73 × 10^4^	–1.100	3007.3	–0.482	6.89 × 10^3^	–0.868	1958.8	–0.511
(2, 2)	6.49 × 10^4^	–1.101	3003.2	–0.486	5.48 × 10^5^	–1.333	4281.1	–0.450
(3, 0)	4.34 × 10^6^	–1.565	5785.5	–0.411	4.93 × 10^5^	–1.333	4280.3	–0.445
(3, 3)	8.07 × 10^3^	–0.869	1973.5	–0.509	6.63 × 10^4^	–1.101	3012.2	–0.481
(4, 0)	4.37 × 10^6^	–1.564	5855.1	–0.409	5.82 × 10^4^	–1.099	3051.9	–0.478
(4, 4)	5.00 × 10^6^	–1.567	5791.7	–0.407	5.00 × 10^6^	–1.567	5792.7	–0.407
(5, 0)	5.78 × 10^4^	–1.100	3047.0	–0.470	4.30 × 10^6^	–1.565	5813.4	–0.404
(5, 5)	5.75 × 10^5^	–1.334	4325.1	–0.438	5.04 × 10^6^	–1.567	5826.8	–0.405

**Table 10:** Total Vibrational Excitation of the Reactants, Δ*E*_vib_ = *E_v_* + *E*_*v*′_ – 2*E*_0_ (kcal mol^–1^), for Each NH­(*v*, 1) + NH­(*v*′, 1) Combination, from the Anharmonic Term Values of NH­(*X* ^3^Σ^–^)­^a^

(*v*, *v*′)	Δ*E*_vib_	(*v*, *v*′)	Δ*E*_vib_
(1, 0)	8.9	(1, 1)	17.9
(2, 0)	17.4	(2, 2)	34.8
(3, 0)	25.5	(3, 3)	50.9
(4, 0)	33.1	(4, 4)	66.1
(5, 0)	40.2	(5, 5)	80.4
(3, 1)^*a*^	34.4		

a Additional energy-matched combination, NH­(3, 1) + NH­(1, 1).

**Figure 12:**
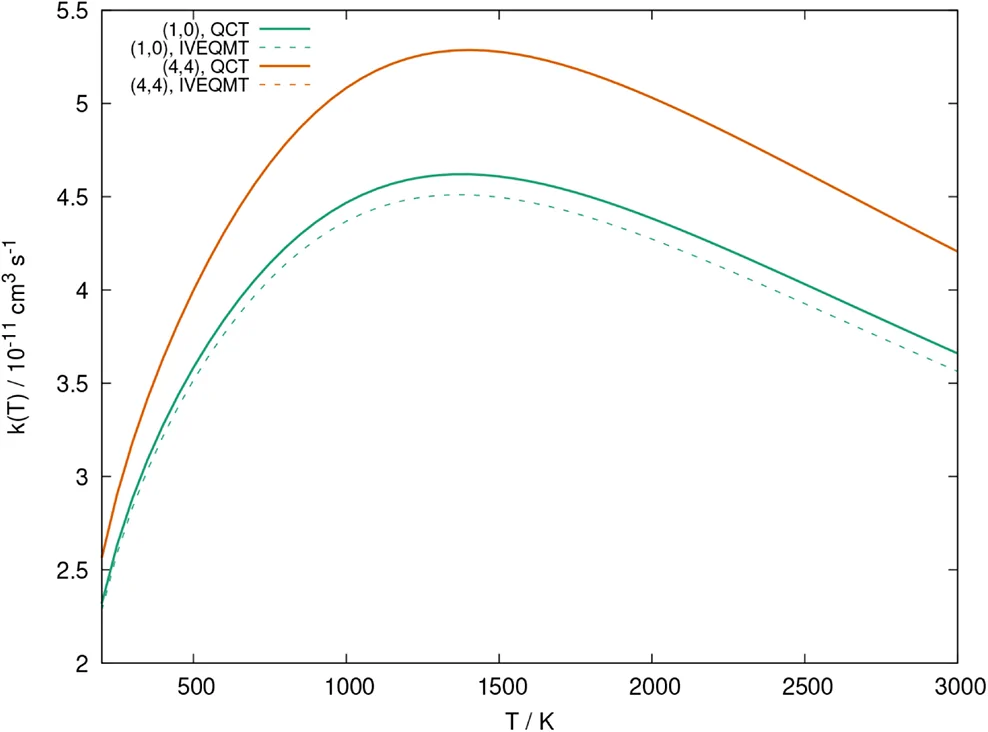
Singlet-surface rate constants *k*(*T*), raw QCT (solid) vs IVEQMT-corrected (dashed), for two representative NH­(*v*, 1) + NH­(*v*′, 1) combinations.

**Figure 13:**
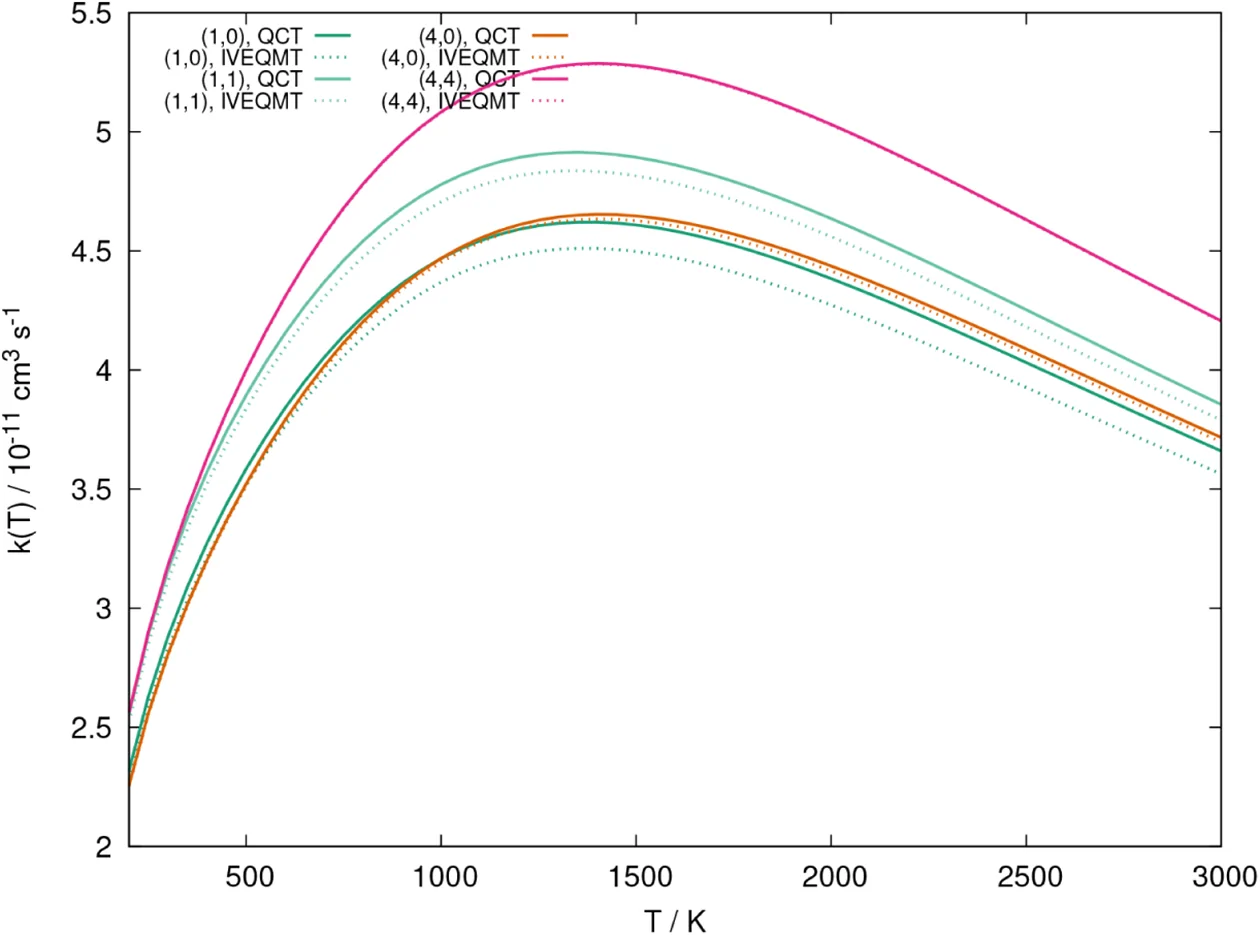
Singlet-surface rate constants *k*(*T*) for selected NH­(*v*, 1) + NH­(*v*′, 1) → N_2_ + H + H combinations, raw QCT (solid) vs IVEQMT-corrected (dotted), showing, for the whole set of four combinations, the same agreement seen in Figure 12.

For completeness we reported in Figure 14 a vibrational-population-weighted average of our ten state-specific rate constants,

2 $$\left\langle k\left(T\right)\right\rangle =\frac{\underset{v,v^{\prime }}{\sum }w_{v}\left(T\right)w_{v^{\prime }}\left(T\right)k\left(T;\;v,\;v^{\prime }\right)}{\underset{v,v^{\prime }}{\sum }w_{v}\left(T\right)w_{v^{\prime }}\left(T\right)}$$

with the Boltzmann weights *w*_*v*_(*T*) computed from the NH­(*X* ^3^Σ^–^) anharmonic vibrational term values (ω_*e*_ = 3282.29 cm^–1^, ω_*e*_ *x*_*e*_ = 78.43 cm^–1^). At thermal equilibrium in this temperature range, the contribution of the vibrationally excited states is relatively small. Moreover, a complete vibrationally averaged rate constant would require the full (*v*, *v*′) grid, including *v* = *v*′ = 0, which the present exploratory data set does not cover; this average is therefore only partial. Figure 14 compares this weighted average (partial) computed from the raw QCT state-specific rate constants with the same average built from the IVEQMT-corrected ones. The trends are the same in the whole temperature range, as expected given the small corrections found at the level of the individual state-specific curves.

**Figure 14:**
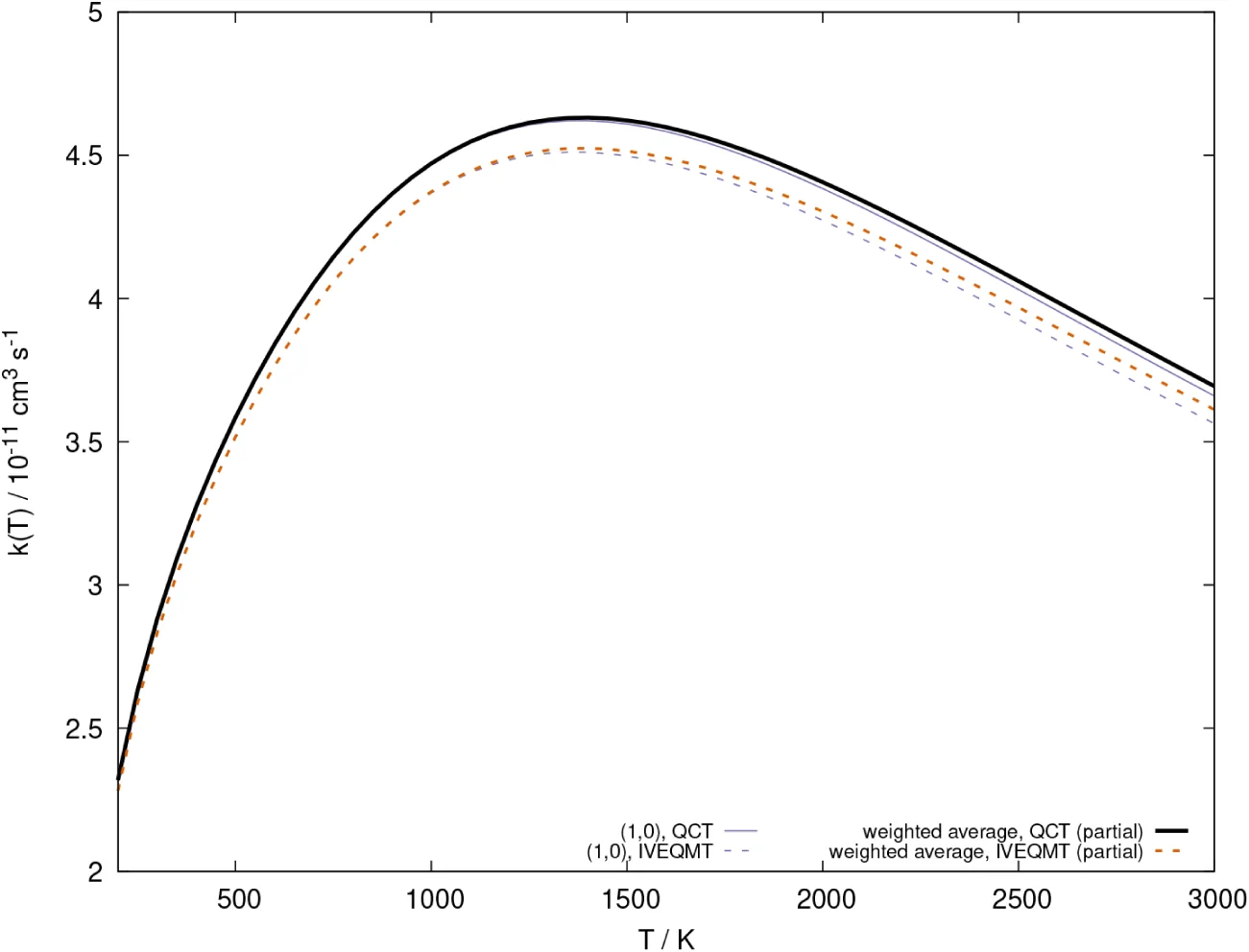
Vibrational-population-weighted average (partial; see text) of the ten state-specific singlet-surface rate constants, from raw QCT (solid black) and IVEQMT-corrected (dashed orange) cross sections. The (1, 0) state-specific curves are shown for reference.

Recently, Qin et al.^46^ performed a comparative QCT study of the OH + CO and OH + SO reactions, revealing how differences in PES topology—specifically, barrier heights and intermediate well depths—lead to contrasting mode-specific reactivity and product energy disposal patterns, with the barrierless OH + SO system exhibiting similar complex-forming dynamics to the NH + NH reaction reported here. From the present study and those of similar systems previously investigated, the vibrational energy content of the nascent product increases markedly with reactant vibrational excitation, demonstrating that efficient funneling of vibrational energy into product degrees of freedom may be a general feature of barrierless or near-barrierless four-atom exchange reactions.

## Conclusions

We have extended our previous QCT study of the NH + NH → N_2_ + H + H reaction to examine the effect of reactant vibrational excitation, for *v* = 1–5 with *j* = *j*′ = 1, in both asymmetric (one reactant excited) and symmetric (both reactants excited) configurations. As in the rotational ground state, the dominant product is N_2_ + H + H, formed via competing one-step and two-step mechanisms. Vibrational excitation modulates the reactivity only modestly compared with the strong suppression previously reported for rotational excitation, confirming the qualitative expectation from the original study’s exploratory calculations. We do find, however, that at low translational energy, states in which both reactants are vibrationally excited show a substantially larger two-step (N_2_H-intermediate) contribution than the corresponding singly excited states. This difference persists for (2, 2) vs (4, 0) at nearly matched total vibrational energy and reverses sign at higher collision energies, whereas the mixed state (3, 1), at the same total energy, has the largest one-step fraction at low collision energy; the low-energy two-step enhancement is thus specific to states in which both reactants are strongly excited. These effects could not be revealed by the narrower grid of the original study. We also find that the vibrational energy content of the nascent N_2_ increases markedly with reactant vibrational excitation. A per-trajectory energy-balance analysis further shows that the title reaction is a source of kinetically hot H atoms, with kinetic energies reaching about 100 kcal mol^–1^ for the most excited combination; this partitioning is itself mechanism-dependent and is systematically more pronounced for the two-step pathway. These three calculated quantities—the one-step/two-step branching ratio, the vibrational energy content of the nascent N_2_, and the thermal rate constants—are, respectively, testable against time-resolved or velocity-map-imaging measurements of the N_2_H intermediate’s lifetime, infrared emission or chemiluminescence measurements of vibrationally excited N_2_, and, once the triplet-surface contribution is also available, existing combustion/pyrolysis kinetic data.^6,10^ Singlet-surface rate constants are also reported for a relatively wide range of temperature. The present results make the N_2_H_2_ DMBE PES a useful testbed for how different forms of internal and translational energy control reactivity in barrierless four-atom exchange reactions. All conclusions refer to the singlet N_2_H_2_ surface; the role of the triplet surface, which carries three times its spin-statistical weight, remains an open question.
